# Development of Immunopathogenesis Strategies to Treat Behçet's Disease

**DOI:** 10.1155/2012/261989

**Published:** 2012-04-03

**Authors:** Osman Köse

**Affiliations:** Department of Dermatology, School of Medicine, Gülhane Military Medical Academy, Tevfik Saglam Street No. 1, 06018 Ankara, Turkey

## Abstract

Behçet disease is a chronic relapsing vasculitis with unclear etiology and immunopathogenesis. Antigenic stimuli, antigen presenting cells, T cells, monocyte, and neutrophil and endothelial cells are major parts of the pathology of the disease. Understanding of the new pathogenic mechanisms based on molecular structure of the disease helps us in improving the novel therapeutic modalities. These drugs target specific and nonspecific inhibition of the immun system. These therapies include biologic agents, new topical and systemic immunosuppressants, tolerizing agents, and immunoablation. Novel treatment will be promising to treat the especially recalcitrant cases to conventional therapy. In this paper, new aspect of the immunopathogenesis of Behçet's diseases and novel treatment modalities will be discussed.

## 1. Introduction

Behçet disease (BD) is a vasculitis that, characterized by recurrent aphthous stomatitis, genital ulcers, skin lesions, relapsing uveitis, articular, neurologic, urogenital, vascular, intestinal, and pulmonary manifestations [1–3]. BD has been reported worldwide but has a distinct geographic distrubition, with highest prevalences in countries such as Turkey, İran, and Japan which are place on silk road. Although much has been learned during recent years on the pathogenesis and treatment of the disease, the etiology and pathogenesis of BD have not been fully clarified [4, 5]. Symptoms of the disease are considered to be based on the correlation between the genetic intrinsic factors and the triggering extrinsic factors, because more than 60% of BD patients are associated with HLA-B 51. Immune-mediated mechanisms play a major role in the pathogenesis of the disease, and inflammatory mediators are also involved [4–6]. Nowadays, recent investigations have made clear explanations about the pathogenesis of the disease. The hypersensitivity of T lymphocytes to different types of antigens plays a crucial role in the pathogenesis [3–6]. 

The present paper overviews an update on the immunopathogenesis of BD and also novel treatment based on pathogenesis. 

## 2. Immunopathogenesis of Behçet's Disease

**Figure 1:**
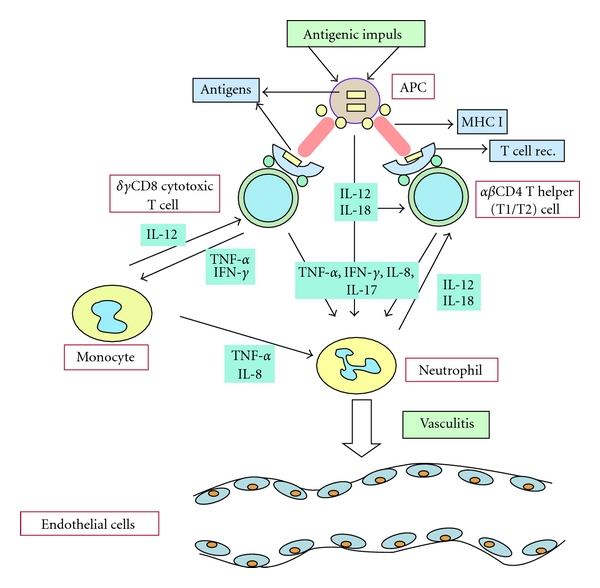
The immunopathogenesis of Behçet's diseases.

BD is an inflammatory disorder characterized mainly by mucocutaneous findings and uveitis. However, it can be present with other cutaneous symptoms such as pseudofolliculitis, erythema nodosum, and pyoderma gangrenosum [2–5]. It can be present with articular, neurological, pulmonary, intestinal manifestations other than classical triad. The close relationship between the genetic and triggering external factors is thought to be present in the pathogenesis of BD [3, 4]. The immunopathogenesis of BD is shown in Figure 1. Critical region for BD in the human major histocompatibility complex (MHC) gene could be pinpointed to a 46 kb segment between the MHC class I gene and HLA-B51 gene [5]. 

### 2.1. Heat Shock Proteins (HSP)

HSP, which essentially scavenge denatured intracellular proteins, are supposed to be induced by microorganisms and mammalian tissues under a variety of stressful conditions, and they may be involved in the pathogenesis of some autoimmune diseases [5]. The serum levels of IgA antibodies to mycobacterial Hsp-65, which cross recats with selected strains of *S. Sanguinis*, are increased significantly. These HSP presented by APCs can directly stimulate *α*, beta T cells, and *γδ*+T cells which play important roles in the oral mucosal immunity as the first defense against microorganisms. Altered expression of Hsp-60 was found in ulcerative lesions of BD and RAS suggesting that HSP-60 has an association with the etiology or chronicity of these inflammatory lesions [7].

### 2.2. APCs (Antigen Presenting Cells)

APC is specialized cell that helps fight off foreign substances that enter the body. Different types of antigens include viruses, bacteria, heat shock protein, and endothelial antigens that stimulate the APC. These cells send out signals to T cells (other immune system cells) when an antigen enters the body [6]. Antigen presentation stimulates T cells to become either “cytotoxic” CD8+ cells or “helper” CD4+ cells. Cytotoxic cells directly attack other cells carrying certain foreign or abnormal molecules on their surfaces. Helper T cells, or Th cells, coordinate immune responses by communicating with other cells. Dysfunction of the APC is responsible for T-cell hypersensitivity. And also this reaction expresses delayed type hypersensitivity mediated by interleukins such as IL-12 and IL-18 in the cutaneous lesions [4].

### 2.3. Neutrophils

These cells are the main elements of the innate immunity. Meanwhile, cytokines and chemokines secreted from APCs and T cells induce the neutrophil hyperactivation. Hyperactivity of the neutrophils is a major part of the immunological abnormalities observed in BD [5, 6, 8]. Activated neutrophils secrete some cytokines which prime themselves and also stimulate Th1 cells. Th1 lymphocytes have a major role in immunopathogenesis of BD [6, 8].

### 2.4. *γδ*+T Cells

*γδ*+T cells, which are important of mucosal immunity have an crucial role. Activity of the BD increases with high level of *γδ*+T cells in circulation and mucosal lesions. CD+8 *γδ*+T cells rather than CD4+ T cells were activated in vivo in Behçet's patients [3, 6, 8]. In our study, expression of the *γδ*+T cells in oral ulcers from BD patients were studied. It was found heterogeneous expression of the *γδ*+T cells throughout the epithelium and in connective tissue around the ulcer base [9].

### 2.5. Proinflammatory Cytokines

It was claimed that tumor necrosis factor (TNF) *α* gene is closely linked to the HLA-B 51 gene, in view of the major role played by this proinflammatory cytokine in BD [3, 6, 8]. An overproduction of proinflammatory cytokines from cellular resources appears to be responsible for the inflammatory reaction in BD, with interferon-*γ*, TNF-*α*, IL-6, IL-8, and IL-12 being higher in patients with BD. T-cell-produced cytokines, including interleukin (IL)-2, tumor necrosis factor (TNF)-*α*, interferon (IFN)-*γ*, IL-12 and IL-18, are elevated and probably contribute to neutrophil and endothelial cell activation. And also IL-12, and IL-18, which are mainly produced by APCs, regulate the neutrophil function and may play important role in the skewing of immune response [4, 8].

### 2.6. Th17 Cells

It was reported a marked increase in Th17 cell numbers and a decreased frequency of CD4 (+) forkhead box protein 3 positive Treg cells in the peripheral blood of patients with active BD [8–12]. Th17/Th1 ratio was elevated in BD patients with uveitis or folliculitis compared to those without it [11]. Th17 cells regulate inflammation via production of distinct cytokines such as IL-17. There is growing evidence that Th17 cells are pathological in many human autoimmune and inflammatory diseases [8–12]. Th17 cells represent a new subset of Th cells, which mainly produce IL-17A-F, IL-22, and TNF-*α*. IL-6 and TGF-ß induce the differentiation of Th17 cells form naive T cells. Hamzoui et al. found high level of TBX 21 (Th1), RORC (Th17) and Foxp3 (Treg) in neuro-BD. They postulated Th1 and Th17 mRNA expressions involving a possible impairment of Treg cells [13].

### 2.7. IL-21

Geri et al. demonstrated the presence of the IL-21 and IL 17-A producing T cells within the cerebrospinal fluid, brain parenchyma inflammatory infiltrates, and intracerebral blood vessels form patients with active BD and central nervous system involvement. The stimulation of CD(+) T cells with IL-21 increased Th17 and Th1 differentiation and decreased the frequency of Treg cells. IL-21 represents a promising target for novel therapy in BD [14]. Jiang et al. found strong association of a single-nucleotide polymorphism of IL-23R with BD. The results suggested that IL-23R is predisposing genotypes for BD [15].

### 2.8. VEGF

In other study, VEGF (vascular endothelial growth factor) was measured in the cerebrospinal fluid in neuro-BD and was found significantly increased. They speculated that VEGF may be associated with the increased percentages of CD4 cell subpopulation [16].

### 2.9. Vitamin D

Serum vitamin D concentrations and BD activity were investigated. Active BD was associated with lower serum vitamin D levels. These results showed that low levels of vitamin D were associated with a decrease in Treg cells and a skewing of the Th1/Th2 balance towards Th1 [17].

### 2.10. Histopathology

 Histopathogenesis of this disease, characterized by systemic perivasculitis, with presence of early neutrophil infiltration, endothelial cell swelling, and fibrinoid necrosis [1–3]. Prominent neutrophil infiltration is seen in all early mucocutaneous lesions. Recurrent aphthous ulceration, skin pathergy reaction, nodular cutaneous lesion, and also ocular lesion show this type of histopathologic pattern [3, 4].

## 3. Novel Treatment of Behçet's Diseases Based on Immunopathogenesis

In general, BD patients have been treated for suppressing the symptoms. Conventional therapeutic approaches suppress the activity of the leucocytes (antiinflammatory) and lymphocytes (immunosuppressive) in T-cell-mediated diseases, for the suppression of the immune system. Generally, BD patients have been treated with the antisymptomatic drugs as follows; immunosuppressants such as nonsteroid anti-inflammatory agents, steroids, colchicine, cyclosporine-A. [1, 3, 4]. Meanwhile, the treatment of BD therapy remains still empirical, but nowadays new insights into BD immunopathogenesis have led to novel therapeutic approaches [18–20]. On the other hand, HSP seems to play an important role in the pathogenesis of BD. The probability of a new therapy for BD should be as the immune tolerance utilizing the peptides of HSP.

### 3.1. Biologic Agents

Clinical and laboratory observations suggested an important role of TNF-mediated process in the pathogenesis of BD [21–25]. During the last ten-year period, 3 licenced TNF antagonists drugs such as infliximab (chimeric anti-TNF-*α*monoclonal antibody), adalimumab (humanized anti-TNF-*α*monoclonal antibody) and etanercept (fusion protein human p75 TNF-*α* receptor IgG1) are increasingly used off-label for patients with BD.

Off-label use of antitumor necrosis factor (TNF) agents for BD is increasing. It was found 88,12 and 13 primary articles on infliximab, etanercept, and adalimumab, reporting on 325, 37, and 28 patients, respectively [21]. Increased levels of TNF, soluble TNF receptors, and TNF-producing cells were found in the peripheral blood of patients with active disease. Among inflammatory cytokine-related genes, TNF blockade reduced expression of IL-1 receptor type 2, interferon *γ* receptors, IL-6, IL-6 receptors, and IL-17 receptors [22]. It was found that infliximab is capable of interfering with *γδ*+T cell function in BD characterized by dysregulated cell-mediated immunity [23]. Overall, the majority of patients treated with either infliximab, etanercept, or adalimumab showed improvement of their mucocutaneous manifestations [21].

### 3.2. Anti-TNF Agents

#### 3.2.1. Infliximab

Infliximab most frequently has been used in BD [24–31]. The dosing regimen for Infliximab was 5 mg/kg IV at weeks 0, 2, 6, and every 8 weeks thereafter [24] and most of these patients were treated with infliximab; remission of oral ulcers, genital ulcers, erythema nodosum, and other skin lesions were noticed in 91%, 96%, 81%, and 77% of them, respectively. A rapid and dramatic improvement of visual acuity and decrease of ocular inflammation starting 24 hours after infliximab was reported [24, 25]. And also long-term effects of repetitive infliximab infusions had positive results regarding the prevention of ocular relapses and tapering of immunosuppressive therapy [25]. Infliximab was used extensively in entero-Behçet, neuro Behçet, and mucocutaneous BD resistant to conventional therapy [26, 27]. Infliximab showed satisfactory results in patients with progressive neuro-BD in different clinical study [25–27]. Experience with infliximab for vascular involvement is limited to case reports. But response to this drug was impressive, with resolution of symptoms within days and improvement of laboratory and imaging findings [29]. But in some cases of BD, TNF blockers are not enough for suppressing the sympoms of the BD. In one series, the combination of infliximab and methotraxate brings about long-term alleviation of entero-BD and excellent tolerability. [31]

#### 3.2.2. Etanercept

Etanercept was administered subcutaneously (SC) in a dose of 25 mg twice a week or 50 mg once a week. Etanercept was found successful in sustaining remission for mucocutaneous findings in significantly more patients than placebo [32–34]. Using the etanercept for ocular involvement was found in small case series. Etanercept was found effective in more than half of patients treated with etanercept [32, 33]. Isolated patients with central nervous system involvement were treated with etanercept with favorable results [32, 34].

#### 3.2.3. Adalimumab

Adalimumab was administered SC as 40 mg every 15 days [21]. Using the etanercept and adalimumab for ocular involvement was found in small case series. Complete remission was achieved in all patients treated with adalimumab. 3 patients with gastrointestinal involvement have been treated successfully with adalimumab [34, 35]. On the other hand, few patients with central nervous system involvement were treated with adalimumab with good results [36]. In large clinical study, a total of 69 patients with BD have been treated with infliximab. But seventeen of these (25%) have been switched to adalimumab for lack or loss of efficacy or infusion reactions. It can be postulated that patients with BD showing a scarce response or adverse events to infliximab may successfully be treated with adalimumab [37].

There is enough published experience to suggest that TNF blockade represents an important therapeutic advancement for patients with severe and resistant, or intolerant, to standard immunosuppresive regimens BD.

#### 3.2.4. Rituximab

Rituximab is a chimeric monoclonal antibody that acts against the specific B cell antigen, CD 20. Rituximab was found effective in retinal vasculitis and ocular manifestations in BD [38, 39]. Twenty patients of with intractable ocular lesions of BD were randomized to a rituximab or cytotoxic drugs such as methotraxate, prednisone, and cyclophosphamide [38]. Rituximab was found effective in ocular lesions of the diseases [38, 39].

#### 3.2.5. Campath 1-H

Campath 1-h is a humanized anti-CD52 antibody. The CD52 antigen is present on lymphocytes and macrophages, but the predominant effect of anti-CD52 antibody therapy (CAMPATH 1-H) is T-cell depletion. Lockwood et al. explored the therapeutic response to lymphocyte depletion with a humanized anti-CD 52 antibody in active BD. This drug will be a potential alternative treatment for refractory BD [40].

#### 3.2.6. Toclizumab

Toclizumab is a humanized anti-interleukin 6 receptor antibody. Toclizumab binds both to soluble and to membrane-bound IL-6 receptor [41, 42]. Evidences showed that IL-6 has a crucial role in the neuro-immunology of neuro-Behçet diseases. Therefore inhibition of IL-6 signaling could be a new therapeutic regimen for Neuro-Behçet diseases [41]. Tocolizumab was used in 47 year old female with recraftory BD. Excellent results were obtained for 1 year. This experience indicates that tocolizumab may constitute a therapeutic option for refractory BD [42].

#### 3.2.7. Gevokizumab

Gevokizumab (XOMA-052) is an Ig G2 humanized monoclonal antibody against human IL-1*β*, for the potential treatment of BD. In future, this drug will be candidate for the treatment of uveitis in patients with the vasculitic diseases such as BD [43].

#### 3.2.8. Rilonacept and Canakinumab

Two new orphan medicines, Rilonacept (Regeneron) and Canakinumab (Ilaris), are a human anti-IL-1*β* monoclonal antibody. Their mode of action are based on the neutralization of IL-1*β* signaling, resulting in supression of inflammation in patients with disorders of autoinflammation. [44, 45]. IL-1*β* is one of the major cytokines implicated in the pathogenesis of many inflammatory-associated diseases. IL-1*β* is, therefore, becoming a focus for the development of new anti-inflammatory drug products [44]. Reports from clinical trials suggest that two drugs was were well tolerated in most patients and no serious adverse efeects were observed [45].

### 3.3. Tolerization Therapy

Heat shock proteins (HSPs) are synthesized when cells are exposed to nonspecific stimuli such as trauma, heat, and infection HSP has played major role in pathogenesis of BD. Tolerance induction has been used for the treatment of autoimmune uveitis [18, 20]. Within HSP-60, the 336–351 sequence has been shown to induce uveitis when administered subcutaneously. Oral administration of the 336–351 peptide linked to recombinant cholera B-toxin B subunit (CTB) was found effective in inhibiting the development of uveitis. There were not observed adverse effects during the therapy. Tolerization could become an appealing therapeutic option because of its lack of side effects and the possibility of the use of other treatment modalities [19, 20].

### 3.4. Immunoablation

Immunoablation with autologous hematopoietic cell transplantation has shown some effectiveness in the treatment of autoimmune diseases. Myeloablative chemotherapy with immunosuppressive drugs followed by autologous transplantation of T-cell-depleted hematopoietic stem cells was found to be safe and effective in BD [18]. Especially in some cases resistant to immunosuppressive drugs, immunoablations could be alternative treatment modalities to control to BD [20].

### 3.5. Other Drugs

#### 3.5.1. Rebamipide

 Rebamipide can be used for oral aphtous ulcers in BD. This drug inhibits free radicals derived from activated neutrophils and decreases the inhibiting inflammatory cytokine [45, 46]. Matsuda et al. used the rebamipide in a multicenter, double-blind, placebo-controlled study 35 patients with BD were randomized 300 mg/day or placebo for 12–24 weeks. In this study, rebamipide was well tolerated and significantly improved the recurrent aphtous stomatitis [46]. In a recent study, Bang et al. used rebamipide plus colchicine versus colchicine in the treatment to BD-like mice. They found that rebamipide helped the function of colchicine to improve the Herpes simplex virus-induced BD symptoms by inhibiting the expression of NADPH oxidase in a vivo mouse model [47].

#### 3.5.2. Immunomodulators

Tacrolimus and pimecrolimus are macrolide antiinflammatory drugs with potent immunosuppressive activity [48–51]. Tacrolimus is used for its capacity to inhibit T-cell cytokines, such as IL-2, IL-4, and TNF-*α*. Oral and topical tacrolimus were used to treat the intestinal and ocular BD [47, 48]. On the other hand, in a clinical trial, topical pimecrolimus cream plus colchicine tablets versus colchicine tablets were used in the treatment of genital ulcers in BD. Pimecrolimus cream shortens the pain duration in genital ulcers [50]. In another clinical study, pimecrolimus versus placebo was used in genital ulcer of BD and also pimecrolimus was found safe and efficient in the treatment of genital ulcers, by accelerating the healing [51].

#### 3.5.3. Mycophenolate Sodium

Mycophenolic acid (MPA) is a potent, selective, and reversible inhibitor of inosine monophospate dehydrogenase and adenosine deaminase. Enteric-coated mycophenolate sodium (EC-MPS), monosodium salt of MPA, allows delayed release of MPA into the small intestine, and it is associated with less adverse effects [52]. Ten patients received enteric-coated formulation of mycophenolate sodium in a standard dose of 720 mg twice daily for six months. Treatment with EC-MPS leads to significant decrease in BD activity. Side effects were mild and did not lead to discontinuation of therapy [53].

#### 3.5.4. Mycophenolate Mofetil

It was presented that 4 cases with parenchymal neuro-BD, where used immunosuppresive drugs could not be continued to intolerance or inefficacy. These patients benefited well from mycophenolate mofetil. The benefit was sustained during 3–7 years of follow-up [54].
